# Cytomegalovirus adrenalitis mimicking adrenal metastasis in an immunocompetent patient

**DOI:** 10.1093/jscr/rjac122

**Published:** 2022-03-26

**Authors:** Jin-soo Park, Matan Ben-David, Catriona McKenzie, Charbel Sandroussi

**Affiliations:** Department of Hepatobiliary and Upper Gastrointestinal Surgery, Royal Prince Alfred Hospital, Sydney, Australia; School of Medicine, University of Notre Dame, Sydney, NSW, Australia; School of Medicine, University of Sydney, Sydney, NSW, Australia; Department of Hepatobiliary and Upper Gastrointestinal Surgery, Royal Prince Alfred Hospital, Sydney, Australia; School of Medicine, University of Sydney, Sydney, NSW, Australia; School of Medicine, University of Sydney, Sydney, NSW, Australia; Department of Tissue Pathology and Diagnostic Oncology, New South Wales Health Pathology, Royal Prince Alfred Hospital, Sydney, Australia; Department of Hepatobiliary and Upper Gastrointestinal Surgery, Royal Prince Alfred Hospital, Sydney, Australia; School of Medicine, University of Sydney, Sydney, NSW, Australia

## Abstract

The adrenal gland is a common site of metastasis due to its rich blood supply. Adrenalectomy is typical treatment in the management of oligometastatic disease. We present an unexpected finding of cytomegalovirus (CMV)-related adrenalitis mimicking adrenal metastasis. A 54-year-old female was reviewed with a history of BRCA2-mutated, hormone receptor-positive invasive ductal cancer of the right breast diagnosed 12 years prior. Surveillance fluorodeoxyglucose positron emission tomography (FDG-PET) demonstrated a new focus of FDG avidity in the left adrenal gland, for which she underwent adrenalectomy. Histopathology revealed CMV-related adrenalitis in an otherwise immunocompetent patient without history of human immunodeficiency virus (HIV) or other immunocompromise. We describe the first case of CMV adrenalitis in a patient without acquired immunodeficiency syndrome. This case was initially presumed to be adrenal metastasis in the context of disseminated metastatic breast cancer and a PET-avid left adrenal lesion.

## INTRODUCTION

The adrenal gland is a common site of metastasis due to its rich sinusoidal blood supply [1], with 13–27% of autopsy studies demonstrating adrenal metastases in disseminated metastatic cancer [2]. In the context of disseminated metastatic disease, imaging concordant with adrenal metastasis can be safely managed with adrenalectomy, with improvements in disease-free survival described in small case series [1, 2]. However, histopathologic assessment may yield unexpected results. We present an unexpected finding of cytomegalovirus (CMV)-related adrenalitis mimicking adrenal metastasis. This case presents a finding that is both unexpected and unprecedented: to our knowledge, there are no reported cases in the literature of CMV-related adrenalitis in patients without acquired immunodeficiency syndrome (AIDS) or cellular immunosuppression.

## CASE REPORT

A 54-year-old female was referred to a tertiary referral surgeon for consideration of left adrenalectomy for presumed adrenal metastasis. The patient had a background of BRCA2-mutated, hormone receptor-positive invasive ductal cancer of the right breast diagnosed 12 years prior, for which she had undergone right mastectomy and axillary lymph node dissection, followed by completion of adjuvant endocrine, radiation and chemotherapy. She had also undergone subsequent radiation therapy for metastases to the brain and lumbar spine, with good response. There was no history of human immunodeficiency virus (HIV), AIDS or lymphopenia. There were no symptoms of CMV infection, and serology was not performed.

**Figure 1:**
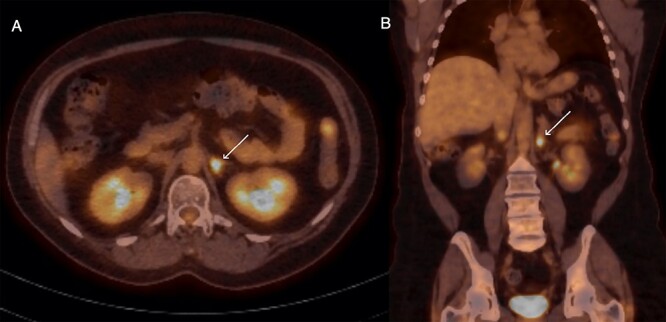
Axial (**A**) and coronal (**B**) sections of FDG-PET demonstrating glucose-avid lesion in the left adrenal (arrow).

Surveillance fluorodeoxyglucose positron emission tomography (FDG-PET) demonstrated a new focus of FDG avidity in the left adrenal gland (Fig. 1). The adrenal lesion was not symptomatic or functional. Magnetic resonance imaging (MRI) of the adrenals did not demonstrate any lesion in the left adrenal gland. The case was discussed, and a joint multi-disciplinary decision was made to proceed with adrenalectomy in lieu of concerns regarding cumulative radiation exposure, and to leave radiation therapy options open in case of future disease recurrence. Pre-operative biopsy was not undertaken due to the risk of complications and due to the fact that a negative result would likely not have changed the course of management, a position echoed by a series reporting a complication rate of 13.6%, and concluding that needle biopsy did not alter clinical management in any of their patients [3].

The patient underwent elective laparoscopic transabdominal left adrenalectomy. There were no intraoperative or postoperative complications, and she was discharged the day after surgery. On subsequent phone follow-up 4 weeks post-operation, the patient was well and reported return to normal function.

**Figure 2:**
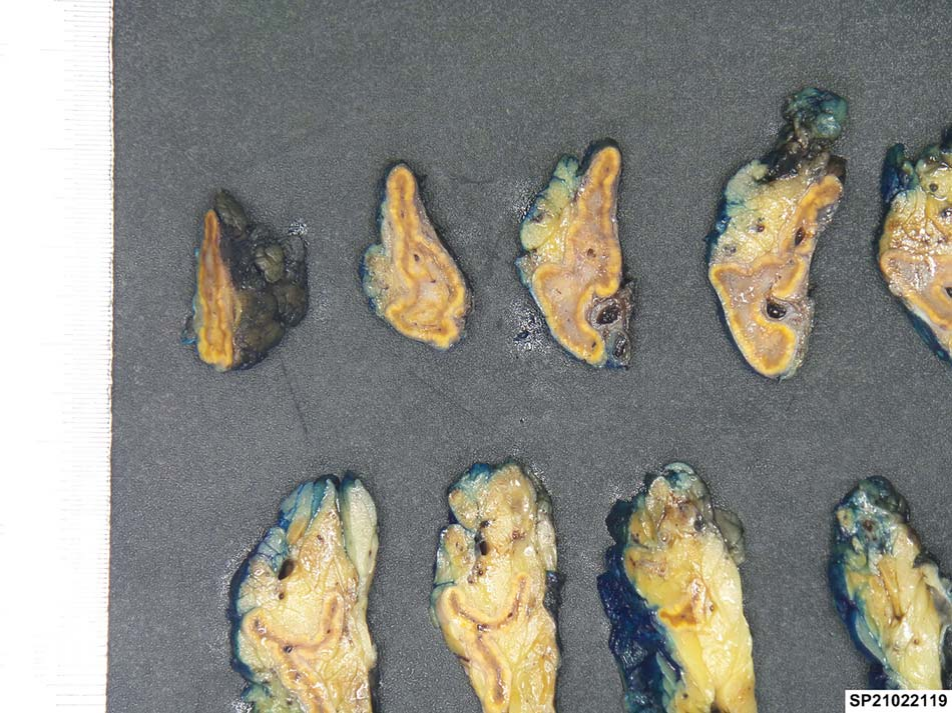
Cut sections of the left adrenal specimen.

**Figure 3:**
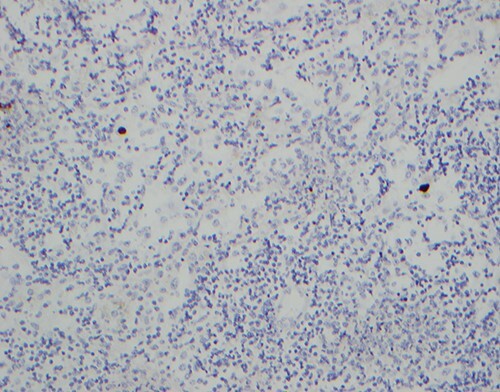
Immunohistochemical stain for CMV, with infected cells (brown) showing characteristic CMV nuclear inclusions.

No mass lesions could be seen on macroscopic examination of the left adrenal specimen (Fig. 2). On microscopic examination, the adrenal medulla was expanded by a lympho-histiocytic infiltrate with microgranulomas noted, as well as scattered cells showing characteristic CMV nuclear inclusions (Fig. 3 ). Immunohistochemical staining for CMV was positive, consistent with a pathological diagnosis of adrenalitis secondary to CMV infection. Other organism stains (Gram, Ziehl Neelson) were negative. No metastatic malignancy was identified.

## DISCUSSION

PubMed and MEDLINE searches confirmed that there are no cases in the literature that describe CMV-related adrenalitis in an immunocompetent patient. This pathology is described in patients with AIDS, with a series of 74 autopsy cases of AIDS demonstrating CMV adrenalitis in 30 (41%), and remarking that the adrenal gland is the most frequently affected organ in AIDS [4]. This sentiment is echoed in another autopsy series of 44 patients with AIDS, where 40 of 73 (55%) adrenal glands were found to have CMV adrenalitis [5].

Outside of concurrent AIDS, CMV-associated adrenalitis has been described in two case reports of renal transplant recipients [6, 7]. In both case reports, the donors were positive for anti-CMV IgG antibodies. Both renal transplant recipients developed adrenal insufficiency, which resolved with administration of ganciclovir.

Another case report of CMV-related adrenal insufficiency not associated with AIDS is described in a patient with Stage IV diffuse large B-cell lymphoma who initially demonstrated clinical remission following completion of eight cycles of rituximab, cyclophosphamide, doxorubicin, vincristine, and prednisone (R-CHOP), but then developed progressive endocrine failure preceding death [8]. Autopsy demonstrated CMV infection of lungs, pancreas, pituitary gland and adrenals.

We describe the first case of CMV adrenalitis in a patient without AIDS. This case was initially presumed to be adrenal metastasis in the context of disseminated metastatic breast cancer and a PET-avid left adrenal lesion. This unexpected finding reinforces that the surgeon must entertain a broad list of differentials when resecting lesions of indeterminate nature.

## CONFLICT OF INTEREST STATEMENT

None of the authors have any financial or professional disclosures.

## FUNDING

None.
